# Preservation of Bone Tissue Integrity with Temperature Control for In Situ SR-MicroCT Experiments

**DOI:** 10.3390/ma11112155

**Published:** 2018-11-01

**Authors:** Marta Peña Fernández, Enrico Dall’Ara, Alexander P. Kao, Andrew J. Bodey, Aikaterina Karali, Gordon W. Blunn, Asa H. Barber, Gianluca Tozzi

**Affiliations:** 1 Zeiss Global Centre, School of Mechanical and Design Engineering, University of Portsmouth, PO1 3DJ, Portsmouth, UK; 2 Department of Oncology and Metabolism and INSIGNEO Institute for in Silico Medicine, University of Sheffield, S1 3DJ, Sheffield, UK; 3 Diamond Light Source, Oxfordshire, OX11 0DE, UK; 4 School of Pharmacy and Biomedical Sciences, University of Portsmouth, PO1 2DT, Portsmouth, UK; 5 School of Engineering, London South Bank University, SE1 0AA, London, UK

**Keywords:** bone, X-ray radiation, tissue damage, SR-microCT, digital volume correlation, temperature control

## Abstract

Digital volume correlation (DVC), combined with in situ synchrotron microcomputed tomography (SR-microCT) mechanics, allows for 3D full-field strain measurement in bone at the tissue level. However, long exposures to SR radiation are known to induce bone damage, and reliable experimental protocols able to preserve tissue properties are still lacking. This study aims to propose a proof-of-concept methodology to retain bone tissue integrity, based on residual strain determination using DVC, by decreasing the environmental temperature during in situ SR-microCT testing. Compact and trabecular bone specimens underwent five consecutive full tomographic data collections either at room temperature or 0 °C. Lowering the temperature seemed to reduce microdamage in trabecular bone but had minimal effect on compact bone. A consistent temperature gradient was measured at each exposure period, and its prolonged effect over time may induce localised collagen denaturation and subsequent damage. DVC provided useful information on irradiation-induced microcrack initiation and propagation. Future work is necessary to apply these findings to in situ SR-microCT mechanical tests, and to establish protocols aiming to minimise the SR irradiation-induced damage of bone.

## 1. Introduction

Bone is a highly heterogenous, anisotropic and hierarchical material that is organised at various levels to optimise its mechanical competence [1]. Thus, it is essential to understand the mechanics of its different components and the structural relationships between them at the different dimensional scales [2,3,4]. This is of fundamental importance since many musculoskeletal pathologies, such as osteoporosis, are associated with alterations in bone quality at the micro- and nanoscale [5]. Therefore, novel techniques aim at characterising the deformation mechanisms of bone in a three-dimensional (3D) manner, from apparent to tissue level, and establishing their links with bone structure [6,7,8].

To date, the only experimental method that allows for 3D strain measurements within the bone structure is digital volume correlation (DVC) in combination with in situ microcomputed tomography (microCT) testing [9,10,11]. DVC has been widely used in bone mechanics to investigate full-field displacement and strain in cortical [12] and trabecular [13,14] bone at different dimensional scales and loading conditions, providing a unique insight to the 3D deformation of such complex material. Nevertheless, in order to characterise bone failure mechanisms at the tissue level, high-resolution microCT is needed [11,15,16]. High-energy synchrotron radiation (SR) microCT has proven to provide fast high-quality image acquisition of bone microstructure with high spatial resolution (~1 µm), and together with in situ mechanical studies, it has allowed for a detailed coupling between 3D bone microstructure and deformation [6,17,18]. Furthermore, recent studies have combined in situ SR-microCT mechanics with DVC to investigate the internal strain and microdamage evaluation of cortical bone [12], trabecular bone [14] and bone-biomaterial systems [19], enhancing the understanding of bone failure at the microscale. 

However, it is known that high exposures to SR X-ray radiation lead to a deterioration in the mechanical properties of bone as a consequence of collagen matrix degradation [20,21]. Similarly, ionising radiation, such as gamma rays, commonly used to sterilise bone allografts [22], and X-rays, negatively affects the mechanical and biological properties of the tissue by the degradation of the collagen present in the bone matrix [20,23,24,25,26,27]. Specifically, radiation produces reactive free radicals by the radiolysis of water molecules, which splits the polypeptides chains of the collagen and induces cross-linking reactions, causing collagen denaturation [28,29,30]. In clinical practice, the adverse effects of gamma radiation during sterilization have been successfully reduced by irradiating the bone while frozen [31,32]. Lowering the temperature is beneficial, as it reduces the mobility of free radicals and, therefore, their ability to interact with collagen molecules [33,34]. Particularly, Hamer et al. [31] observed that cortical bone irradiated at low temperatures (−78 °C) was less brittle and had less collagen damage when compared to the bone irradiated at room temperature. Additionally, Cornu et al. [32] showed that ultimate strength, stiffness and work to failure were not reduced significantly on trabecular bone irradiated under dry ice. In the field of high-resolution X-ray imaging of biological samples, protection against radiation damage is also essential to preserve their integrity. Cryofixation methods have been demonstrated to protect biological samples from visible structural damage and have enabled cryo-soft X-ray tomography (cryo-SXT) to become the only imaging modality able to provide nanoscale 3D information of whole cells in a near-native state [35,36,37]. However, soft X-rays (~0.1–1 keV) are not able to penetrate bone tissue, nor can they be accommodated for in situ mechanics protocols. Furthermore, cryotechniques involve freeze-drying of the specimens at −150 °C and have been shown to induce microdamage and significantly reduce torsional strength, compressive yield stress and compressive modulus of cortical bone [32,38,39,40]. Hence, low temperatures positively influence bone preservation during irradiation. However, mechanical testing of bone in such conditions, below the freezing temperature of water, cannot be conducted, as the mechanical properties of bone would be affected. In fact, due to the large water content of bone, ice crystals may cause structural damage to the tissue [23].

Therefore, it is essential to define some guidelines in order to preserve bone tissue integrity and mechanics during in situ SR-microCT experiments. Very recently, DVC applied to SR-microCT images of trabecular bone was used to investigate the influence of SR irradiation-induced microdamage on the bone’s apparent mechanics [14]. Microcracks were detected in the bone tissue after long exposures to SR radiation, despite the apparent elastic properties remaining unaltered. Also, high local strain levels were observed that corresponded to the microdamaged areas. However, reducing the total exposure to SR X-ray radiation was able to preserve bone integrity and plasticity. The results of that study [14] provided important information on bone degradation and residual strain accumulation resulting from SR X-ray exposure, but the study had some limitations. Firstly, bone specimens were subjected to cyclic mechanical loading during SR-microCT imaging; thus, the full-field strain measurements were not entirely due to SR irradiation but also to the mechanics. In fact, DVC results showed that even at reduced exposures to SR radiation, there were some regions of high strain concentration, which may have been induced by the mechanical load and further enhanced by the irradiation. Secondly, reducing the total exposure by decreasing the exposure time per projection during SR-microCT acquisition notably decreased image quality and, consequently, DVC performance. Hence, further evaluation and optimisation of the imaging setup is needed in order to preserve bone integrity while maximising image quality for reliable DVC-computed full-field measurement within the bone tissue.

In this context, there is a clear need to define experimental protocols for in situ SR-microCT mechanics able to preserve bone tissue integrity against SR X-ray radiation-induced damage, exploiting the research conducted in different fields. The aim of this study is, therefore, to propose a novel proof-of-concept methodology to retain bone tissue integrity, based on residual strain determination via DVC, by decreasing the environmental temperature during SR-microCT testing.

## 2. Materials and Methods

### 2.1. Specimen Preparation

Samples were obtained from a fresh bovine femur. A section (20 mm in thickness) was cut with a hacksaw from the proximal diaphysis of the femur and a diamond-coated core drill was used to extract 4 mm cylindrical compact (n = 2) and 6 mm trabecular (n = 2) bone specimens under constant water irrigation. The ends of the cores were trimmed to achieve a 12 mm length for the compact and a 16 mm length for the trabecular bone specimens. Brass endcaps were used to embed the ends of the specimens (~2 mm), ensuring perpendicularity between the bone cores and the endcap bases. Samples were kept frozen at −20 °C and thawed for approximately 2 h in saline solution at room temperature before imaging.

### 2.2. SR-MicroCT Imaging

**Figure 1:**
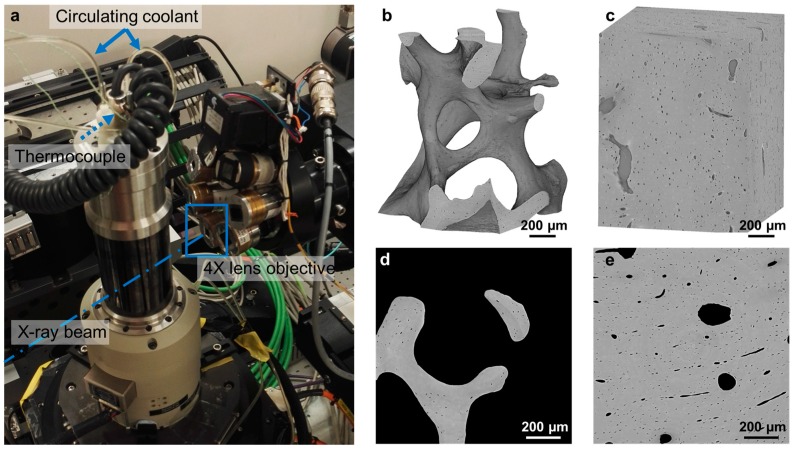
(**a**) Experimental setup at I13-2 beamline. The direction of the beam is indicated by the dashed-dotted line. Specimens were scanned within a loading device using a 4× lens objective. The temperature in the device was controlled with a circulating coolant and monitored on the tissue via an additional thermocouple attached to the surface of the specimens. SR-microCT reconstructed volume of interest (VOI) (1.13 × 1.13 × 1.46 mm^3^) analysed for (**b**) trabecular and (**c**) compact bones with an effective voxel size of 0.81 µm. Two-dimensional cross-section through the middle of the VOI after masking the bone marrow (**d**) from the trabecular bone and the Haversian and Volkmann’s canals (**e**) from the compact bone.

SR-microCT was performed at the Diamond-Manchester Imaging Branchline I13-2 (Figure 1a) of Diamond Light Source (DLS), Oxfordshire, UK. A partially coherent polychromatic ‘pink’ beam (5–35 keV) of parallel geometry was generated by an undulator from an electron storage ring of 3.0 GeV. The undulator gap was set to 5 mm for data collection and, to limit bone damage, 11 mm for low-dose alignment. The beam was reflected from the platinum stripe of a grazing-incidence focusing mirror and high-pass filtered with 1.4 mm pyrolytic graphite, 3.2 mm aluminium and 50 µm steel. The propagation (sample-to-scintillator) distance was approximately 40 mm. Images were recorded by a sCMOS (2560 × 2160 pixels) pco.edge 5.5 (PCO AG, Kelheim, Germany) detector which was coupled to a 500 µm-thick CdWO_4_ scintillator and a visual light microscope with a 4× objective lens, providing a total magnification of 8×. This resulted in an effective voxel size of 0.81 µm and a field of view of 2.1 × 1.8 mm^2^. A total of 1801 projection images were collected over 180° of continuous rotation (‘fly-scan’), with an exposure time of 512 ms per projection (11 ms overhead per exposure), adopting the imaging conditions reported in [14]. The total scanning time was approximately 15 min. The projection images were flat-field- and dark-field-corrected prior to image reconstruction using SAVU [41], which incorporated ring artefact suppression and optical distortion correction [42]. Each specimen underwent five full consecutive tomographic data collections.

### 2.3. In Situ Testing and Temperature Control

Specimens were placed within an in situ testing device (CT5000-TEC, Deben, Bury Saint Edmunds, UK) and kept in saline solution during image acquisition (Figure 1a). The device is equipped with a 5 kN load cell, Peltier heated and cooled jaws with a temperature range from −20 °C to +160 °C and an environmental chamber. A small preload (2–5 N) was first applied to ensure good end-contact and avoid motion artefacts during tomographic acquisition, after which the actuator was stopped, and the jaws’ positions held throughout the test. Bone specimens (N = 1 compact and N = 1 trabecular) were imaged at room temperature (T_room_ ≈ 23 °C) and at ~0 °C (N = 1 compact and N = 1 trabecular) by cooling and keeping the Peltier jaws at the target temperature. A thermocouple (Type K, RS Pro, RS Components, Corby, UK) was also attached to the surface of the bone samples and was used during the in situ test to monitor the temperature directly at the tissue during image acquisition and between tomographies. Temperature measurements and recordings were processed with a thermocouple data logger (USB TC-08, Pico Technology, St Neots, UK). For reliable temperature measurements, the thermocouple was calibrated prior to the experiment.

### 2.4. Image Post-Processing

Five datasets were obtained for each specimen and further processed using Fiji platform [43]. After image reconstruction, each 3D dataset consisted of 2000 images (2400 × 2400 pixels) with 32-bit grey-levels. Images were converted to 8-bit greyscale and cropped to parallelepipeds (volume of interest (VOI)) with a cross-section of 1400 × 1400 pixels (1.134 × 1.134 mm^2^) and a height equal to 1800 pixels (1.46 mm) in the centre of the scanned volume (Figure 1b,c). Noise in the images was reduced by applying a nonlocal means filter [44], where the variance of the noise was automatically estimated for each dataset [45]. The five consecutive scans per specimen were first rigidly registered using the first acquired dataset as a reference. The 3D rigid registration was based on sum of squares differences as a similarity measurement between the reference and each target image. Finally, the filtered VOIs were masked by setting to zero-intensity the non-bony voxels (i.e., Haversian and Volkmann’s canals in compact bone and bone marrow space in trabecular bone). A binary image (value of one for bone voxel and zero elsewhere) was first created using Otsu’s threshold algorithm followed by a despeckling filter to remove 3D regions less than three voxels in volume both in white and black areas, which are mainly related to nonfiltered noise. Additionally, isolated pixels were removed, and small holes were filled by using a series of morphological operations as described in [16]. The quality of the binary images was checked by visual inspection. Masked images, with the original greyscale value in the bony voxels and zero elsewhere, were obtained by multiplying the filtered image with the final binary image (Figure 1d,e).

### 2.5. Digital Volume Correlation

Digital volume correlation (DaVis v10.0, LaVision, Göttingen, Germany) was carried out to evaluate the residual strain in the bone tissue due to progressive damage induced by X-ray exposure to SR radiation during SR-microCT at different temperatures. DaVis software is based on a local approach of correlation, which has been widely used in bone mechanics [13,14,46]. Details on the operating principles of the software are reported elsewhere [16,47]. DVC was applied to the masked images to avoid large strain artefacts in regions with insufficient greyscale pattern (i.e., bone marrow) [16]. A different multi-pass scheme was used for the DVC computation on compact and trabecular specimens after an evaluation of the baseline strains in the first two consecutive tomograms for the four specimens, obtained in a nominal ‘zero-strain’ state, where the irradiation-induced damage was considered minimal (Supplementary Materials). A final subvolume of 32 voxels, reached via successive (predictor) passes using subvolumes of 112, 56, 48 and 40 voxels, was used for the compact bone, whereas, for the trabecular bone, a final subvolume of 64 voxels, reached via successive passes of 112, 88, 80 and 72 voxels, was adopted. Given the voxel size of the SR-microCT images, the final DVC measurement spatial resolution was 25.9 µm for compact and 51.8 µm for trabecular bones. Additionally, in both cases, subvolumes with a correlation coefficient below 0.6 were removed from the resultant displacement vectors to avoid artefacts due to poor correlation. The different processing schemes for both bone typologies mainly depended on the higher number of features (i.e., osteocyte lacunae) available in the compact bone specimens compared to the trabecular ones, which allowed a smaller subvolume size to be used for the former [11]. To evaluate the 3D full-field residual strain distribution in the bone tissue over time in relation to the damage induced by continuous X-ray exposure to SR radiation, DVC was performed by registering the reference image (first acquired tomogram) with each of the remaining tomograms. First (ε_p1_) and third (ε_p3_) principal strains and maximum shear (γ_max_) strain were computed within the bone volume after a bicubic interpolation of the measured strain. Furthermore, in order to couple the initiation and propagation of microcracks in the tissue with the displacement and first principal strain directions, dedicated MATLAB (v2018a, MathWorks, Natick, MA, USA) scripts were developed. The MATLAB scripts allow for the representation of any set of orthogonal slices within the volume and for the computation of the displacement and first principal strain values and their corresponding direction for each subvolume.

## 3. Results

### 3.1. In Situ Testing and Temperature Control

**Figure 2:**
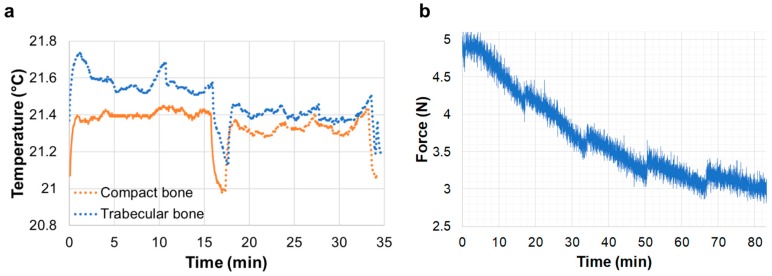
(**a**) Temperature readings measured using a thermocouple attached to the compact and trabecular bone surface at room temperature. The solid line corresponds to thermocouple readings during ~15 min with the X-ray shutter opened and the thermocouple in the beam path. Dotted lines represent thermocouple readings during tomographic acquisition. The sudden drop and consequent rise in temperature coincide with the closing and opening of the X-ray shutter. (**b**) Force readings in trabecular bone specimen at room temperature during five consecutive tomograms. An increase in the force was observed and corresponded with the opening of the X-ray shutter.

Temperature readings from the thermocouple attached to the surface of the bone specimens suggested a consistent temperature gradient (∆T = 0.4 °C) at each exposure period (Figure 2a) corresponding to the opening (rise in temperature) and closing (drop in temperature) of the X-ray shutter. Small fluctuations in the temperature were recorded once the X-ray shutter was open, as they are more evident during tomographic acquisition compared to the steady position. However, those fluctuations were far less important than the temperate gradients recorded between consecutive tomographies. The stress-relaxation curves recorded during in situ testing showed that the X-ray beam significantly influenced the relaxation behaviour of the trabecular bone specimen at room temperature (Figure 2b). A consistent increase in the force was recorded after the start of each tomographic acquisition. This trend was not observed for the compact bone specimen.

### 3.2. Compact Bone

**Figure 3:**
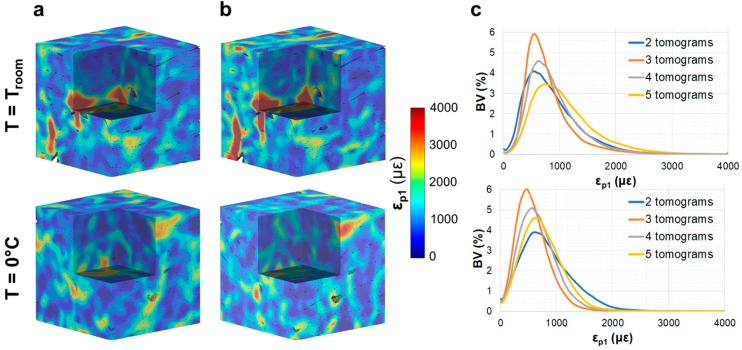
Three-dimensional first principal strain (ε_p1_) distribution in compact bone tissue imaged at room temperature (top) and 0 °C (bottom) after two (**a**) and five (**b**) acquired tomograms. A representative cube (~1 mm^3^) in the centre of the analysed VOI is represented. Histograms of the residual strain distribution (**c**) in the tissue are shown for all the acquired tomograms.

**Figure 4:**
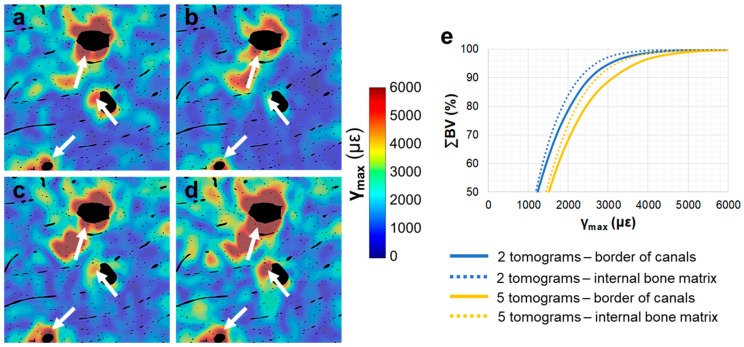
Maximum shear strain (γ_max_) distribution in compact bone tissue imaged at room temperature. Cross-sections in 2D are shown after (**a**) two, (**b**) three, (**c**) four and (**d**) five acquired tomograms. Arrows indicate highly strained regions. A cumulative histogram of the residual strain (**e**) in the tissue voxels around the canals (solid lines) and the remaining bone matrix (dotted lines) is shown for after two and five tomograms.

No damage was visually detected in the compact bone specimens after five tomograms, either at room temperature or 0 °C. The residual ε_p1_ distribution (Figure 3) did not show any notable changes in the tissue after the acquisition of two (Figure 3a) and five (Figure 3b) tomograms, with some localised areas of higher residual strain in the specimen imaged at room temperature. The strain histograms (Figure 3c) showed peak values below 1000 µε for both specimens, and no clear trends were observed between exposure to SR radiation and peak strain values. However, histograms showed tails with higher strains after five tomograms at room temperature compared to 0 °C. Similar findings were observed for the residual ε_p3_ and γ_max_ (Figure S2), suggesting a strain redistribution between consecutive tomographies, which did not cause important damage overall. Highly strained regions of the specimen tested at room temperature were localised around the Haversian and Volkmann’s canals (Figure 4). Residual strain in a region of approximately 20 µm surrounding the canals was compared to the strain in the internal bone matrix volume. Particularly, the cumulative histograms of γ_max_ (Figure 4e) after two and five acquired tomograms showed slightly higher strains around the canals for the same bone volume percentage.

### 3.3. Trabecular Bone

**Figure 5:**
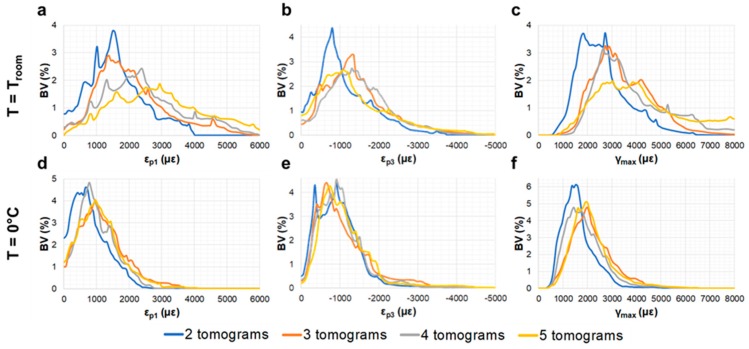
Histograms of the residual strain distribution in trabecular bone tissue imaged at room temperature (top) and 0 °C (bottom). (**a**,**d**) First principal strains (ε_p1_), (**b**,**e**) third principal strains (ε_p3_) and (**c**,**f**) maximum shear strains (γ_max_) after each acquired tomogram are shown.

**Figure 6:**
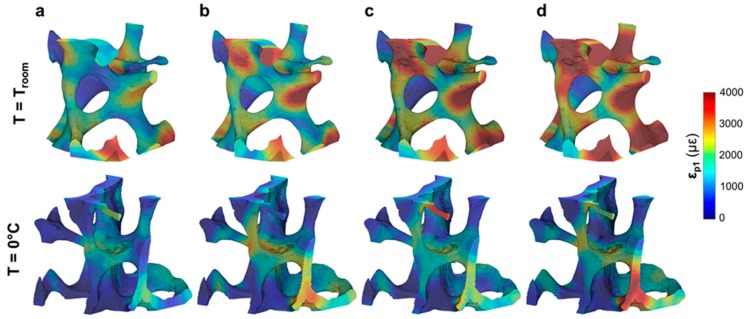
Three-dimensional full-field first principal strain (ε_p1_) distribution in trabecular bone tissue imaged at room temperature (top) and 0 °C (bottom) after the acquisition of (**a**) two, (**b**) three, (**c**) four and (**d**) five consecutive tomograms. A representative cube (~1 mm^3^) in the centre of the analysed VOI is represented.

A visual inspection of the reconstructed images showed the presence of several microcracks after five tomograms, corresponding to ~80 min of total exposure to SR X-ray radiation, in the trabecular bone specimen at room temperature. However, decreasing the temperature to 0 °C facilitated tissue preservation, as microdamage was not observed. Furthermore, the high levels of residual strain measured with DVC correlated well with the microdamage visible from the images. The histograms of residual strain distributions (Figure 5) after each tomogram highlighted the differences between the two trabecular bone specimens. On one hand, the specimen imaged at room temperature showed a consistent increase in residual strain when increasing the exposure to X-ray radiation (Figure 5a–c). This trend was clearly observed in ε_p1_ (Figure 5a), for which strain peak values increased from ~1500 to ~3000 µε after two and five consecutive scans, respectively. ε_p3_ (Figure 5b) peak values were found to be below −1500 µε, whereas peak γ_max_ (Figure 5c) ranged from ~2000 µε to ~3500 µε after two and five tomograms, respectively. The residual strain accumulation was less evident for the trabecular bone specimen maintained at 0 °C (Figure 5d–f). In fact, peak strain values remained below ±1000 µε for ε_p1_ (Figure 5d) and ε_p3_ (Figure 5 e), respectively, and below 2000 µε for γ_max_ (Figure 5f) after five tomograms. The 3D full-field strain distribution in the trabecular bone (Figure 6) was accumulated in the tissue after each tomogram. In particular, for the specimen at room temperature (Figure 6, top), it could be seen that ε_p1_ was increasing after each tomography, and regions of high residual strains after two full tomographies (Figure 6a, top) were progressively enlarged, reaching strain values of over 4000 µε after five tomograms (Figure 6d, top). This strain accumulation was less pronounced in the specimen at 0 °C (Figure 6, bottom), although some areas of high strain concentration were observed after each tomogram. Furthermore, some strain redistributions could be seen after three (Figure 6b, bottom) and four (Figure 6c, bottom) full tomographies.

### 3.4. Tracking of Crack Formation

**Figure 7:**
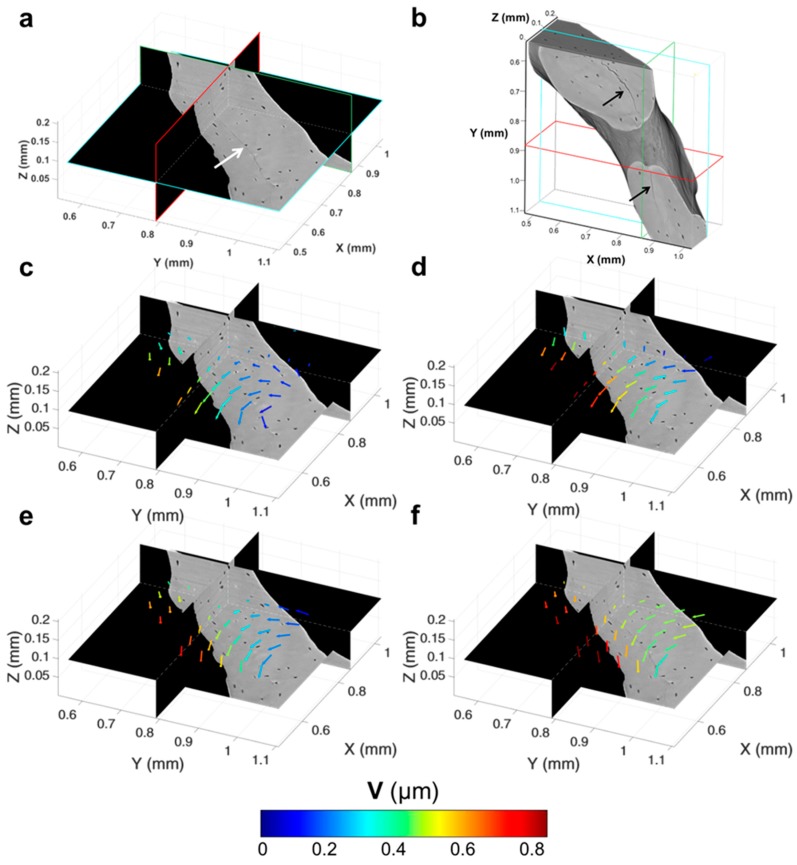
Microcrack tracking in trabecular bone tissue imaged at room temperature. (**a**) Representative orthoslices and (**b**) 3D representation of the trabecular bone region tracked over time. Arrows indicate the microcracks visible in the tissue. (**c**–**f**) Digital volume correlation (DVC)-computed displacement field (V) in each subvolume on the analysed region of interest around a microcrack at different time points corresponding to the acquisition of (**c**) two, (**d**) three, (**e**) four and (**f**) five tomograms. Vector lengths are identical, and the colour code refers to the V magnitude in micrometres.

**Figure 8:**
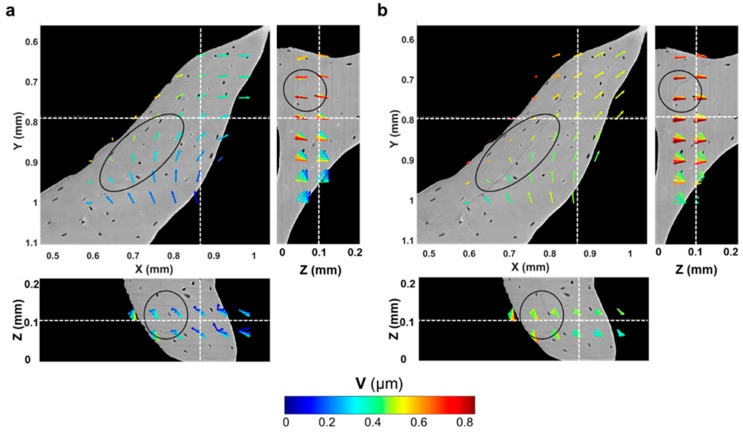
DVC-computed displacement field through the region of interest analysed around the microcracked area (**a**) before (fourth tomogram) and (**b**) after cracking was visible (fifth tomogram). Oval regions highlight damaged areas of bone tissue. Vector lengths are identical, and the colour code refers to the displacement vector length (V) in micrometres.

**Figure 9:**
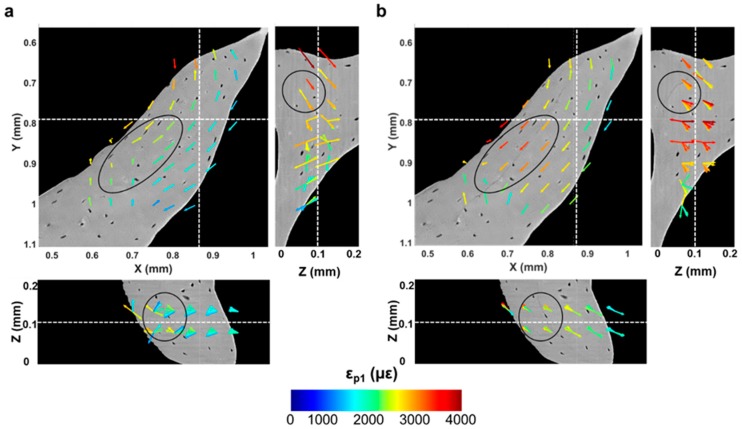
DVC-computed first principal strain (ε_p1_) through the region of interest analysed around the microcracked area (**a**) before (fourth tomogram) and (**b**) after cracking was visible (fifth tomogram). Vectors indicate first principal strain directions in each subvolume. Oval regions highlight damaged areas of bone tissue, which correspond to high orientation changes in the principal strain direction before and after cracking. Vector lengths are identical, and the colour code refer to the εp1 magnitude.

**Figure 10:**
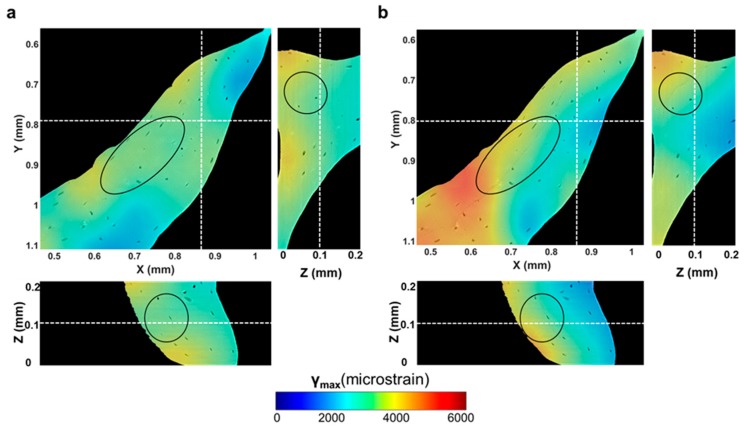
DVC-computed maximum shear strain (γ_max_) through the region of interest analysed around the microcracked area (**a**) before (fourth tomogram) and (**b**) after cracking was visible (fifth tomogram). Oval regions highlight damaged areas of bone tissue, which correspond to an increase in shear strain values before and after cracking. High discontinuities in shear strains were identified in the damage region (**b**), which may suggest the direction of crack propagation.

Microcracks were clearly visible in the trabecular bone specimen imaged at room temperature after five tomograms (Figure 7a,b). A region inside a trabecula (Figure 7b) was tracked during the in situ test to couple the residual strain accumulation with the crack formation. The displacement field around the damaged region (Figure 7c–f) suggested a relative motion between regions at both sides of the cracks since the earliest stages, before cracking was visible (Figure 7c–e). In fact, low displacements were found on one side, and those were mainly directed toward the positive z-direction, whereas, in the neighbouring side, displacements were progressively increased and reoriented toward the negative z-direction. After cracking (Figure 7f), displacements further increased around the crack, and a pronounced reorientation of their direction was observed. A deeper look at the displacement in the orthogonal planes (Figure 8), before and after crack formation, evidenced the discontinuities in the displacement field in proximity to the crack. Particularly, before crack formation (Figure 8a), displacement showed a high misorientation in the XY and XZ planes. After cracking (Figure 8b), the displacement field at one end of the crack was found perpendicular to the crack direction (XY plane), whereas it seemed aligned with the crack on the other end, which may indicate the further propagation direction. Both ε_p1_ and γ_max_ showed a progressive increase in the microcracked region, reaching values above 4000 µε for ε_p1_ (Figure 9b) and approximately 5000 µε for γ_max_ (Figure 10b) in the damaged area. In general, tensile strains were the most correlated to microdamage detection. In fact, the directions of ε_p1_ (Figure 9) suggested a combination of tensile and shear modes of crack formation. In addition, the principal directions before cracking seemed to be highly disordered throughout the analysed volume. In particular, the highlighted vectors before cracking (Figure 9a) exhibited a very abrupt change in orientation, whereas the same areas after cracking (Figure 9b) were considerably aligned with the microcrack. γ_max_ (Figure 10) increased after crack formation, and discontinuities at both sides of the crack were observed (Figure 10b). Moreover, higher shear strain levels were found at one side of the crack (XY plane), which also corresponded to principal strains and displacements perpendicular to the crack, thus possibly suggesting the direction of crack propagation.

## 4. Discussion

The proof-of-concept experiment reported herein enabled important understanding of the SR X-ray radiation-induced damage to the integrity of bone tissue. The residual strain accumulation caused by SR X-ray radiation was quantified for the first time using DVC applied to in situ SR-microCT images, and the effect of the environmental temperature on the SR irradiation-induced damage in bone tissue was addressed. It is known that irradiation has a deleterious effect on the structural and mechanical properties of bone as a result of collagen matrix degradation due to the formation of collagen cross-links and eventual rupture of the collagen fibres [20,27]. Several studies have addressed the effect of high-energy SR X-ray radiation on the mechanical properties of bone [20,21,26,48], and safe dose values (35 kGrays) were defined to preserve bone mechanics [20]. However, during in situ SR-microCT studies, a reduction of the dose is related to a reduction in the total exposure to SR radiation and, therefore, the signal-to-noise ratio of the acquired tomograms, with a consequent reduction in image quality and increased DVC errors [14]. Therefore, new protocols need to be defined in order to preserve bone tissue while maintaining good image quality. Furthermore, whether bone integrity can be preserved by controlling the temperature during in situ SR experiments still remains unexplored.

The overall change in temperature during image acquisition was minimal (∆T = 0.4 °C) (Figure 2a) and in line with previous reports on SR beam heating [49,50]. Wallander and Wallentin [51] showed that X-ray-induced heating can lead to significant temperature increase (i.e., nanowire at 8 °C above room temperature) at typical synchrotron beamline fluxes. As a strategy for reducing the X-ray-induced heating, it was suggested to improve the heat transfer of the target material to the surroundings, for example, by immersing the samples in liquid [51]. However, it still remains unclear whether that thermal gradient in a very short period of time (opening/closing of the beam shutter) may induce collagen degradation. As specimens were held between the loading stage platens during the in situ test, the effect of the X-ray beam on the stress-relaxation behaviour of the specimens could be observed (Figure 2b), similar to the data reported in [52]. With only a fixed preload applied, an increase in the load was identified for the trabecular bone specimen at each cyclic period that corresponded with the opening of the X-ray shutter. Both trabecular and compact bone exhibit a highly viscoelastic behaviour; however, this is more evident for trabecular bone due to the large content of bone marrow in its cavities. Thus, the loadcell of the loading stage was not accurate enough to capture any changes in the stress-relaxation behaviour for the compact bone specimen. Heat causes a transformation of the collagen molecule, known as the collagen shrinkage phenomenon [53], whereby the collagen molecule develops a contractile force that is held constant [54,55] at a given temperature (shrinkage temperature). This shrinkage behaviour is related to the cross-links in the collagen and its stability [53]. Even though the specimens in the current study were kept at a constant temperature (~23 °C), the beam-induced temperature rise of 0.4 °C may contribute to the activation of a similar contractile force, which is a clear indicator of the harmful effects of the SR irradiation on bone tissue.

The results obtained from the current study have shown that reducing the temperature to 0 °C notably reduced the irradiation-induced microdamage and residual strain in trabecular bone specimens (Figure 6). However, minimal effect was observed for compact bone (Figure 3). Nguyen et al. [30] reported that the mechanical properties of compact bone were decreased by a lower dose than that affecting trabecular bone. However, it has been shown here (Figure 3) that the structural integrity of compact bone tissue was not compromised, as microcracks were not detected as in the trabecular bone tissue. In any case, specimens were not mechanically tested; thus, whether the regions of high strain concentration found in compact bone (Figure 3) influence the mechanical properties is still unknown. Furthermore, Peña Fernández et al. [14] showed that the presence of microcracks was not always related to changes in the apparent elastic properties of the irradiated bone.

Although the overall residual strain in compact bone imaged at room temperature was low, with peak strain values below 1000 µε for ε_p1_ (Figure 3), some highly strained regions were identified in close proximity to Haversian and Volkmann’s canals (Figure 4). Canals and osteocyte lacunae are known to act as stress concentrating features in specimens subjected to mechanical load [6,12]; however, the effect of irradiation on these specific sites has never been considered. Haversian canals contain unbound water [56], and as ionising radiation produces the release of free radicals via radiolysis of water molecules [29], it is expected that a larger number of free radicals, which could interact with the collagen and induce cross-linking reactions, are found in proximity to the canals due to the higher water content. 

Lowering the environmental temperature to 0 °C had a positive effect on the DVC-measured residual strain in trabecular bone, which showed a peak principal strain value below 1000 µε (Figure 5); furthermore, no microdamage was visually detected on the reconstructed tomograms. These results are consistent with medical studies on the effect of gamma irradiation, where it was shown that irradiating bone specimens while frozen did not affect the mechanical properties of bone [31,34]. In fact, decreasing the temperature reduces the mobility of the water, and, therefore, decreases the mobility of highly reactive oxygen free radicals produced by high-energy X-ray radiation. Impairing that mobility protects the collagen by reducing cross-linking reactions within its molecules [57,58]. The effect of freezing on the mechanical properties of bone has been previously studied [59,60,61,62] and no statistical differences were found after freezing, nor after several freeze-thaw cycles [63,64]. It should be noted that, during the proposed experiment, specimens were immersed in saline solution at 0 °C, and ice crystals, which may cause structural damage to the tissue [63], were not observed at any stage of the experiment. 

The irradiation-induced damage in the trabecular bone imaged at room temperature resulted in microcracks that were visible in the tissue even if the specimen was not subjected to any mechanical load. At the nanoscale, SR irradiation-induced free radical attack of the collagen network results in a cross-linking reaction that degrades the structural integrity of the collagen fibres [20,29,30]. Previous studies using atomic force microscopy have shown that crack formation and bone fracture occur between the mineralised collagen fibrils. Fantner et al. [65] proposed that the mineralised collagen fibres are held together by a nonfibrillar organic matrix that acts as a glue. The glue resists the separation of the mineralised collagen fibrils, avoiding the formation of cracks, when a load is applied to the bone. During the formation of microcracks, work that stretches the glue molecules would be required to separate the mineralised collagen fibrils. Irradiation may affect that mechanism by damaging the sacrificial bonds, as a result of the observed shrinkage behaviour, which could lead to the rupture of those bonds after prolonged exposure to irradiation and consequent microcrack formation. At the macroscale, DVC-computed displacements (Figure 7) suggest a vortex motion around the microcracked region, which results in a shrinkage process of the material and the formation of a microcrack that follows an unusual pattern in fracture mechanics. The denaturation of the collagen may not be homogeneous throughout the bone tissue; therefore, crack propagation would follow the degeneration process of the collagen.

DVC was successfully used to understand crack formation and propagation in bone. Christen et al. [12] investigated the initiation and propagation of microcracks in cortical bone using DVC; however, full-field displacements and strains were only evaluated in terms of magnitude, but the directions were not explored. Additionally, specimens were pre-cracked before mechanical testing; thus, crack initiation and propagation was expected around the notch region. In this study, microcracks were not induced by mechanical loading, but by SR irradiation instead. Discontinuities in the displacement field (Figure 8a) corresponded to high-orientation changes in the strain field (Figure 9a) that could indicate crack formation. Furthermore, perpendicularity of displacement (Figure 8b) and principal strains (Figure 9b) to the crack might be related to a crack propagation front. Similar crack formation mechanisms were observed in clay deformation using digital image correlation (DIC) following desiccation [66,67]. Like the results herein reported, in opening mode, the direction of the crack was perpendicular to that of ε_p1_, whereas, for cracks in mixed opening-sliding mode, ε_p1_ was found parallel to the direction of the crack (Figure 9b). Those studies [66,67] concluded that cracks formed a network which is found after thermal shocks, and the authors emphasized the need to develop a multiscale approach to better understand crack formation and propagation. Similar to those findings, irradiation-induced microcracks need to be further investigated at different dimensional levels to properly understand the formation mechanisms.

This study has some limitations. First, only one specimen per bone type was tested at each temperature, and the mechanical properties of the bone specimens were not evaluated after irradiation. Residual strain maps suggested that a decrease in the temperature had a beneficial effect on preserving bone integrity and mechanics, but specimens were maintained far below physiological conditions (~37 °C); thus, it could be argued that the mechanical properties of bone tissue could have been altered. Further analyses are needed to properly assess the effect of the environmental temperature during in situ SR-microCT experiments, translating the findings of the proposed methodology to in situ SR-microCT bone mechanics. Moreover, a combination of techniques at different dimensional scales would enhance the knowledge of the irradiation-induced damage in bone tissue.

## 5. Conclusions

The 3D full-field residual strain distribution of compact and trabecular bone subjected to high-energy SR irradiation was computed using DVC applied to SR-microCT images acquired at different temperatures. Lowering the temperature during irradiation to only 0 °C had a positive effect on trabecular bone tissue, which—unlike such bone imaged at room temperature—did not present visible microcracks, and residual strain values were not increased with further radiation. However, a minimal effect was observed in compact bone. A shrinkage behaviour induced by both the beam-induced temperature and high-energy irradiation may well be the source of the irradiation-induced damage and microcracks in bone tissue. DVC applied to high-resolution SR-microCT images has proven to be a useful tool for understanding crack formation and propagation in bone tissue. Further work is needed to clearly establish protocols for the application of SR-microCT to the in situ mechanics of bone and potentially extend the knowledge to other biological tissues in order to minimise SR irradiation-induced damage.

## Supplementary Materials

The following are available online at http://www.mdpi.com/1996-1944/11/11/2155/s1, Figure S1: Relationship between (**a**) MAER and (**b**) SDER with the sub-volume size for the four bone specimens, Figure S2: Histograms of the residual strain distribution in compact bone tissue imaged at room temperature (top) and 0 °C (bottom). (**a**) Third principal strains (ε_p3_) and (**b**) maximum shear strains (γ_max_) after each acquired tomogram are shown, Table S1: Random errors for the three displacement components for compact and trabecular bone specimens. Median values of the two specimens per group are shown.
