# Supplementary material for: Preservation of Bone Tissue Integrity with Temperature Control for In Situ SR-MicroCT Experiments
Source: Materials (Basel). 2018 Nov 1;11(11):2155. doi: 10.3390/ma11112155 (PMC6266162; doi:10.3390/ma11112155)
Supplement: Supplementary file 1 [file materials-11-02155-s001.pdf]

Supplementary Materials

Marta Peña Fernández [1], Enrico Dall'Ara [2], Alexander P. Kao [1], Andrew J. Bodey [3], Aikaterina Karali [1], Gordon W. Blunn [4], Asa H. Barber [1,5] and Gianluca Tozzi [1,*]

Evaluation of 'baseline' strains

1. Methods

The evaluation of the baseline strains was performed in the first two consecutive datasets for the four specimens, where irradiation-induced damage was deemed as minimal. As the images were acquired in the same deformed state (i.e. 'zero-strain' repeated scans), null displacement and strain fields are expected. Therefore, any non-zero values of the measured displacement and derived strains using DVC were considered as error. Ten multi-pass schemes [1] with final sub-volume sizes ranging from 8 to 80, in steps of 8 voxels were investigated. For each sub-volume, three different parameters were computed.

- Random errors of the displacements: standard deviation of each displacement component, as in [2].
- Mean absolute strain value: average of the average of the absolute values of the six components of the differential strain, similar to MAER or "accuracy", as in [3].
- Standard deviation of the strain value: standard deviation of the average of the absolute values of the six components of the differential strain, similar to SDER or "precision", as in [3].

2. Results

The random errors of each component of the displacement never exceeded 0.30 μm for the compact bone specimens and 0.33 μm for the trabecular bone specimens (Table S1). The errors obtained for the displacements in the compact bone were higher than those for the trabecular bone in x and y directions, but lower in z direction. A trend could be observed for both bone type specimens where the higher the sub-volume size, the lower the random errors.

Table S1. Random errors for the three displacement components for compact and trabecular bone specimens. Median values of the two specimens per group are shown.

Multi-pass scheme sub-volume sizes (voxels)	Displacement random errors (μm)					
	Compact bone			Trabecular bone		
	X	Y	Z	X	Y	Z
**64-32-24-16-8**	0.30	0.27	0.12	0.32	0.33	0.26
**80-40-32-24-16**	0.25	0.24	0.08	0.25	0.26	0.19
**96-48-40-32-24**	0.23	0.23	0.07	0.23	0.24	0.18
**112-56-48-40-32**	0.23	0.23	0.07	0.20	0.21	0.17
**128-64-56-48-40**	0.22	0.22	0.07	0.17	0.18	0.17
**144-72-64-56-48**	0.22	0.22	0.06	0.16	0.16	0.17
**160-80-72-64-56**	0.21	0.22	0.06	0.13	0.16	0.15
**178-88-80-72-64**	0.21	0.22	0.06	0.13	0.16	0.15
**192-96-88-80-72**	0.20	0.21	0.06	0.13	0.15	0.14
**192-112-96-88-80**	0.20	0.21	0.06	0.12	0.15	0.14

As expected from previous studies on bone [3,4], the strain uncertainties of the DVC had decreasing trends with respect to the sub-volume size, and the values of the mean value of the strain (MAER) were larger than the standard deviation (SDER) (Figure S1). The MAER ranged between 3000 με and 100 με for the compact bone samples and between 5500 με and 300 με for the trabecular

bone samples, in sub-volumes of 8 to 80 voxels (6.5 to 65 μm). The SDER ranged between 1250 με and 30 με for the compact bone and between 5000 με and 140 με for the trabecular bone, in the same sub-volumes.

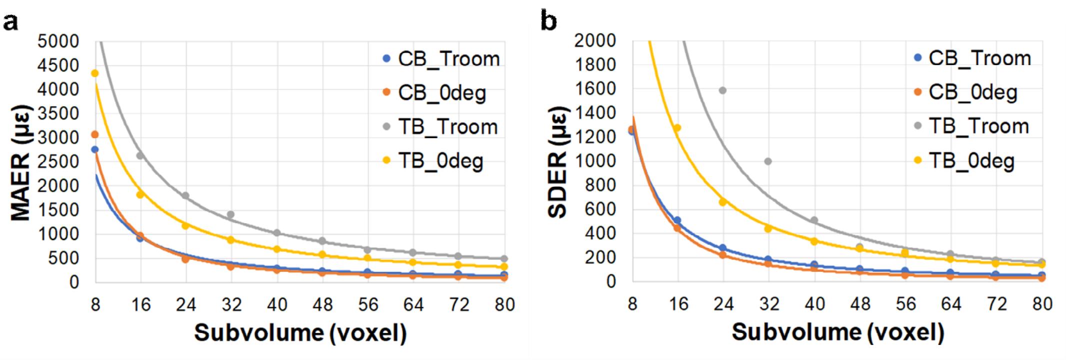

Figure S1. Relationship between (**a**) MAER and (**b**) SDER with the sub-volume size for the four bone specimens.

References

1. Peña Fernández, M.; Barber, A.H.; Blunn, G.W.; Tozzi, G. Optimisation of digital volume correlation computation in SR-microCT images of trabecular bone and bone-biomaterial systems. *J. Microsc.* **2018**, *00*, 1–16, doi: 10.1111/jmi.12745.
2. Palanca, M.; Tozzi, G.; Cristofolini, L.; Viceconti, M.; Dall'Ara, E. 3D Local Measurements of Bone Strain and Displacement: Comparison of Three Digital Volume Correlation Approaches. *J. Biomech. Eng.* **2015**, *137*, 1–14, doi:10.1115/1.4030174.
3. Palanca, M.; Bodey, A.J.; Giorgi, M.; Viceconti, M.; Lacroix, D.; Cristofolini, L.; Dall'Ara, E. Local displacement and strain uncertainties in different bone types by digital volume correlation of synchrotron microtomograms. *J. Biomech.* **2017**, *58*, 27–36, doi:10.1016/j.jbiomech.2017.04.007.
4. Dall'Ara, E.; Peña-Fernández, M.; Palanca, M.; Giorgi, M.; Cristofolini, L.; Tozzi, G. Precision of DVC approaches for strain analysis in bone imaged with μCT at different dimensional levels. *Front. Mater.* **2017**, *4*, 31, doi:10.3389/fmats.2017.00031.

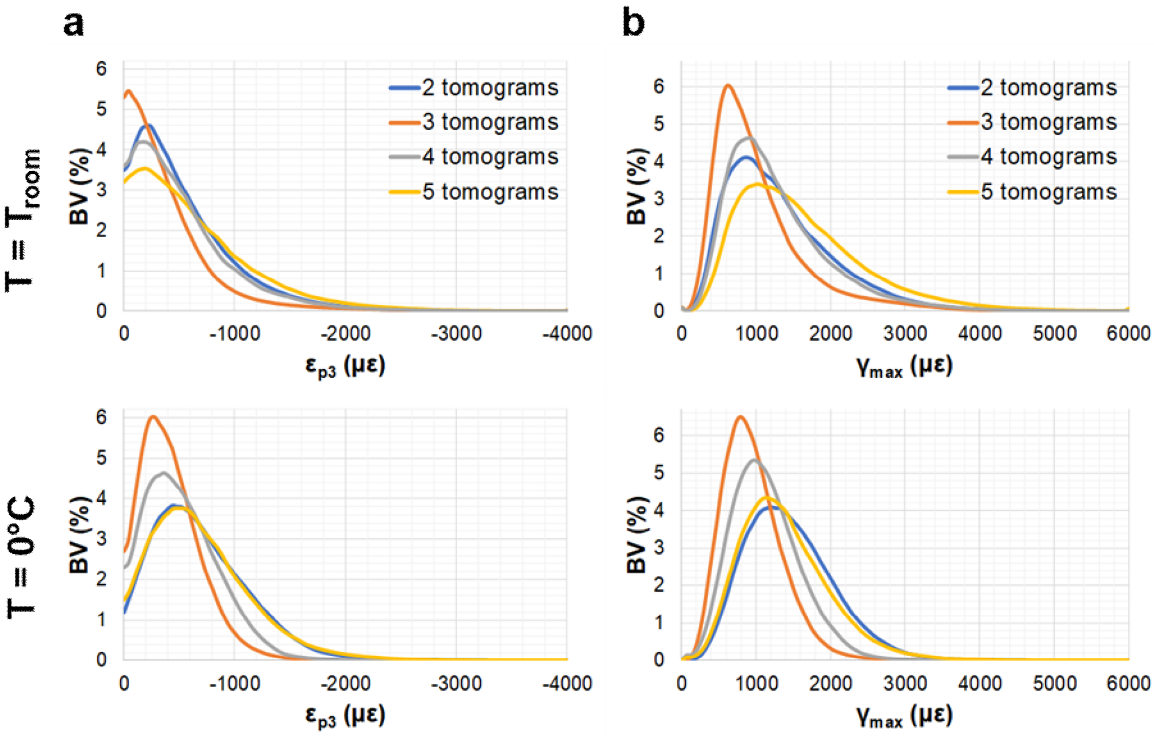

Figure S2. Histograms of the residual strain distribution in compact bone tissue imaged at room temperature (top) and 0 °C (bottom). (**a**) Third principal strains (ε_{p3}) and (**b**) maximum shear strains (γ_{max}) after each acquired tomogram are shown.
